# The correlation and level of agreement between end-tidal and blood gas pCO_2 _in children with respiratory distress: a retrospective analysis

**DOI:** 10.1186/1471-2431-9-20

**Published:** 2009-03-12

**Authors:** James M Moses, Jamin L Alexander, Michael SD Agus

**Affiliations:** 1 Department of Medicine, Children's Hospital Boston, 300 Longwood Ave, AU-522, Boston, MA 02115, USA

## Abstract

**Background:**

To investigate the correlation and level of agreement between end-tidal carbon dioxide (EtCO_2_) and blood gas pCO_2 _in non-intubated children with moderate to severe respiratory distress.

**Methods:**

Retrospective study of patients admitted to an intermediate care unit (InCU) at a tertiary care center over a 20-month period with moderate to severe respiratory distress secondary to asthma, bronchiolitis, or pneumonia. Patients with venous pCO_2 _(vpCO_2_) and EtCO_2 _measurements within 10 minutes of each other were eligible for inclusion. Patients with cardiac disease, chronic pulmonary disease, poor tissue perfusion, or metabolic abnormalities were excluded.

**Results:**

Eighty EtCO_2_-vpCO_2 _paired values were available from 62 patients. The mean ± SD for EtCO_2 _and vpCO_2 _was 35.7 ± 10.1 mmHg and 39.4 ± 10.9 mmHg respectively. EtCO_2 _and vpCO_2 _values were highly correlated (r = 0.90, p < 0.0001). The correlations for asthma, bronchiolitis and pneumonia were 0.74 (p < 0.0001), 0.83 (p = 0.0002) and 0.98 (p < 0.0001) respectively. The mean bias ± SD between EtCO_2 _and vpCO_2 _was -3.68 ± 4.70 mmHg. The 95% level of agreement ranged from -12.88 to +5.53 mmHg. EtCO_2 _was found to be more accurate when vpCO_2 _was 35 mmHg or lower.

**Conclusion:**

EtCO_2 _is correlated highly with vpCO_2 _in non-intubated pediatric patients with moderate to severe respiratory distress across respiratory illnesses. Although the level of agreement between the two methods precludes the overall replacement of blood gas evaluation, EtCO_2 _monitoring remains a useful, continuous, non-invasive measure in the management of non-intubated children with moderate to severe respiratory distress.

## Background

With the advent of capnography, physicians have been given a tool to non-invasively assess the ventilatory status of their patients. This has had far reaching implications in patient care. End-tidal CO_2 _(EtCO_2_) measurement has become standard for clinical monitoring of both adult and pediatric patients undergoing general anesthesia, and has proven to be useful in a variety of other clinical settings.[1,2] In the pre-clinical setting, EtCO_2 _monitoring has been standard of care for patients requiring cardiopulmonary resuscitation and emergency cardiovascular care since 2000. [3-5] In both pediatric intensive care unit (PICU) and emergency department (ED) settings, capnography is now widely used to confirm appropriate endotracheal tube placement and for the continuous management of mechanical ventilation. [6-8] EtCO_2 _monitoring is also useful in identifying apnea and bronchospasm in non-intubated children undergoing procedural sedation [9-13] and in assessing the degree of metabolic acidosis in various pediatric populations. [14-17]

Though EtCO_2 _monitoring has proven to be efficacious in diverse clinical areas, its utility in non-intubated patients with pulmonary disease remains undefined. In patients with significant pulmonary disease, it is generally believed that EtCO_2 _values will not accurately reflect blood gas pCO_2 _because of ventilation-perfusion mismatch, increased dead space, and/or increased shunt fraction. [18-21] In fact, a number of studies have demonstrated the inaccuracy of capnography in intubated and non-intubated patients with pulmonary disease.[20,22-25] However, most of these studies focused on patients with severe lung disease or used technology that is now considered out of date.

Because of the general assumption that EtCO_2 _monitoring is less accurate in patients with pulmonary disease, there is a paucity of data assessing its utility as a corollary to blood gas pCO_2 _in non-intubated pediatric patients with moderate to severe respiratory distress. In this study, we investigated the association of EtCO_2 _to vpCO_2 _in hospitalized non-intubated children with moderate to severe respiratory distress secondary to asthma, bronchiolitis, or pneumonia. We also examined the level of agreement between vpCO_2 _and EtCO_2 _to determine if EtCO_2 _could replace blood gas evaluation in the management of non-intubated pediatric patients with respiratory distress secondary to a pulmonary process.

## Methods

We performed a retrospective chart review of pediatric patients admitted with moderate to severe respiratory distress secondary to asthma, bronchiolitis, or pneumonia to the intermediate care unit (InCU) at Children's Hospital Boston (CHB) between July, 2003-February, 2005. The InCU is designed for patients who are moderately to critically ill, who need close monitoring and increased nursing needs, but who do not need invasive monitoring, acute ventilatory support, or vasopressor therapy. Continuous nasal cannula EtCO_2 _monitoring is standard of care for all patients admitted to the InCU with respiratory distress. The study was approved by the institutional review board of CHB.

All patients admitted to the InCU with moderate to severe respiratory distress secondary to above diagnoses and who had a blood gas evaluation and an EtCO_2 _measurement within 10 minutes of each other were eligible for inclusion. Moderate to severe respiratory distress was defined as tachypnea and oxygen saturation < 94% on room air with retractions and decreased aeration on physical examination. Patients with chronic pulmonary disease (cystic fibrosis, chronic lung disease), cardiac disease, poor tissue perfusion (defined as capillary refill greater than 2 seconds), or underlying metabolic abnormalities were also excluded. Patients undergoing acute respiratory failure defined as immediate subsequent transfer from the InCU to the intensive care unit for invasive respiratory support were also excluded.

Data collected on each patient included patient demographics, vitals measurements, and O_2 _requirement at time of EtCO_2 _reading. The patients' pulmonary status including severity of retractions and level of aeration as recorded by InCU nurses on InCU data tracking flowsheet were also included. Retractions and aeration were measured on a scale ranging from 0 (no retractions, normal aeration, respectively) to 3 (significant subcostal and suprasternal retractions, severely decreased aeration throughout lung fields, respectively). EtCO_2 _values were measured using Microstream^® ^Capnoline™ H nasal cannula (Oridion Medical, Jerusalem, Israel) attached to Invivo MDE Escort Prism^® ^monitors (Invivo MDE, Orlando FL, USA).

### Statistical Analysis

The association between EtCO_2 _and vpCO_2 _values was analyzed using the Pearson product-moment correlation coefficient (r) and simple linear regression. Multivariate linear regression was used to assess for variation in bias between EtCO_2 _and vpCO_2 _according to clinical and patient characteristics. Bland-Altman analysis was performed to determine the level of agreement between EtCO_2 _and vpCO_2 _values.[26,27] We also compared the linear regression between EtCO_2 _and vpCO_2 _to the line of unity between the two measurements to further define the relationship between EtCO_2 _and vpCO_2_. Statistical analysis was performed using SAS Version 9.1 (SAS Institute Inc., Cary, NC, USA).

## Results

There were 80 paired EtCO_2_-vpCO_2 _values from 62 patients. 40 of the 80 paired measurements were simultaneous. The mean ± SD time difference between measurements was 0.67 ± 8.19 minutes. The bias between EtCO_2_-vpCO_2 _values was not significantly affected by the time difference between measurements (p = 0.6110).

Age ranged from 5.5 months-20 years with a median age of 5.7 years. Asthma was the admitting diagnosis for 41 patients, bronchiolitis for 9 patients and pneumonia for 12 patients. Other characteristics of our study sample are included in Table 1.

**Table 1:** Characteristics of Study Sample (n = 62)

Age, median (range), years	5.7 (0.047–20.09)
Diagnosis, n (%)	
*Asthma*	41 (66)
*Bronchiolitis*	9 (15)
*Pneumonia*	12 (29)
RR, mean ± SD, breaths/min	38.3 ± 18.6
Supplemental O_2 _requirement, n (%)	
*Room air*	13 (21)
*O2 req.*	45 (72)
*Not recorded*	4 (6)
Disposition, n (%)	
*Home*	9 (15)
*General Floor*	44 (71)
*ICU*	7 (11)
*Other*	2 (3)
EtCO_2_, mean ± SD, mm Hg	35.7 ± 10.1
vpCO_2_, mean (± SD), mm Hg	39.4 ± 10.9

**Figure 1:**
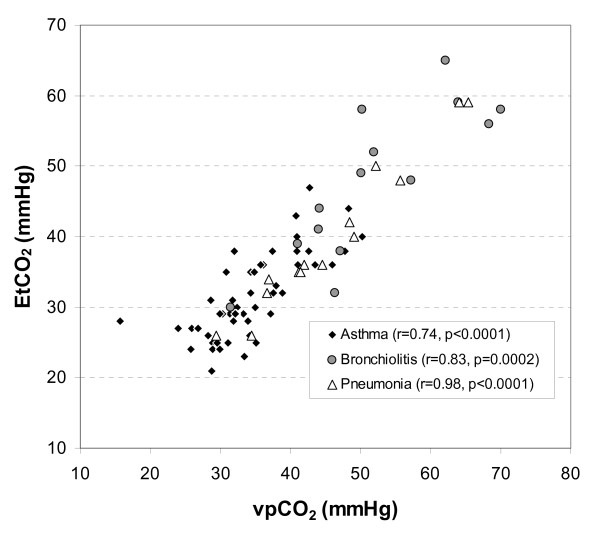
**EtCO_2 _vs. vpCO_2 _by Admitting Diagnosis**.

The mean ± SD for EtCO_2 _and vpCO_2 _was 35.7 ± 10.1 mmHg and 39.4 ± 10.9 mmHg respectively. EtCO_2 _and vpCO_2 _were highly correlated (r = 0.90, p < 0.0001). As shown in Figure 1, the correlations for asthma, bronchiolitis and pneumonia were 0.74 (p < 0.0001), 0.83 (p = 0.0002) and 0.98 (p < 0.0001) respectively.

**Figure 2:**
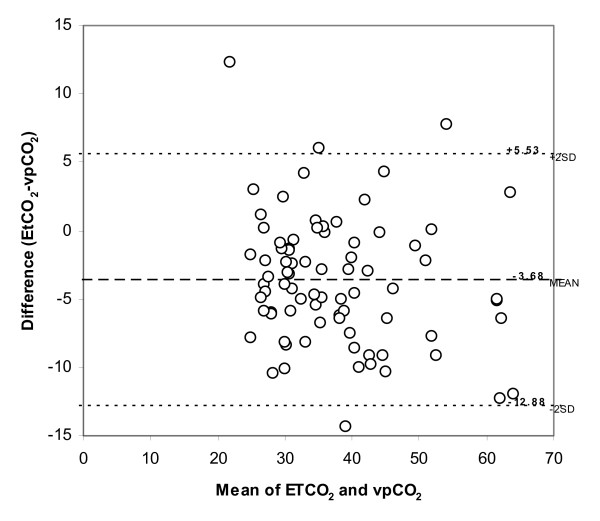
**Bland Altman Plot**.

The mean bias ± SD between EtCO_2 _and vpCO_2 _values from the Bland-Altman analysis (Figure 2) was -3.68 ± 4.70 mmHg. The 95% limits of agreement between EtCO_2 _and vpCO_2 _ranged from -12.88 to +5.53 mmHg. EtCO_2 _was within ± 5 mmHg of vpCO_2 _in 46 of the 80 values and within ± 10 mmHg of vpCO_2 _in 73 of the 80 values. Bias did not significantly vary across the range of averaged values. From multivariate regression, the bias between EtCO_2 _and vpCO_2 _did not vary according to age (p = 0.8445), respiratory rate (p = .9305), oxygen requirement (p = 0.4222), or clinical assessment measurements such as aeration (p = 0.3876) or retractions (p = 0.4381).

**Figure 3:**
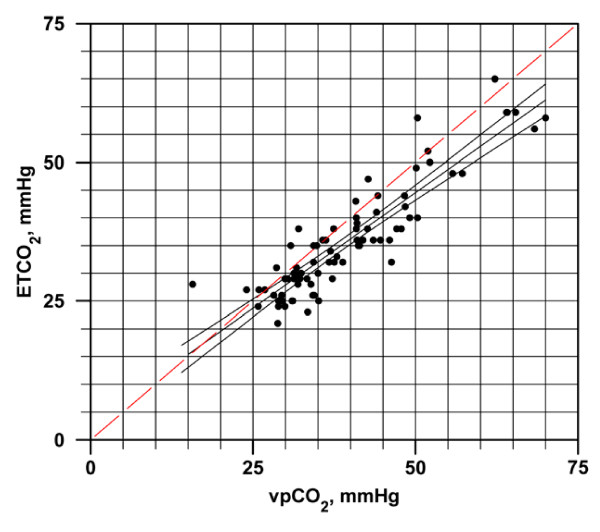
**Regression of EtCO_2 _and vpCO_2 _Values with Line of Unity**. The equation for the regression line is EtCO_2 _= .832904(vpCO_2_) + 2.905448, r^2 ^= 0.8161.

Further analysis was conducted in order to identify the range of EtCO_2 _values in which accuracy would be maximized in relation to vpCO_2_. Figure 3 illustrates the regression of EtCO_2 _and vpCO_2_, which deviated significantly from the line of unity (F(2,78) = 35.5, p < 0.001). The regression line and the line of unity were within 3 mmHg of each other for vpCO_2 _values under 35 mmHg but parted increasingly in the higher range, differing by 5 mmHg above vpCO_2 _of 47 mmHg. This suggests that maximum EtCO_2 _accuracy is achieved when vpCO_2 _is 35 mmHg or below.

## Discussion

The utility of EtCO_2 _monitoring in the assessment and management of non-intubated pediatric patients with moderate to severe respiratory distress has remained largely undefined. Historically, studies have found EtCO_2 _monitoring to have limited accuracy in both intubated and non-intubated patients with pulmonary disease.[20,22-25] Because of these prior studies most physicians agree that in the clinical setting of respiratory distress, EtCO_2 _monitoring is mostly useful in following the trend in ventilatory status and not as a specific correlate to blood gas pCO_2_.[23,28] However, knowing EtCO_2 _can serve as a direct corollary to blood gas pCO_2 _in patients without pulmonary disease[22,25,29], it raises the question of a possible threshold of pulmonary disease that until reached EtCO_2 _remains an accurate tool to assess blood gas pCO_2_.

In this study, we found EtCO_2 _to be highly correlated with venous pCO_2 _and that this relationship was significant across the range of common respiratory illnesses that cause moderate to severe respiratory distress in children. Only one prior study has evaluated the accuracy of non-invasive capnography in non-intubated pediatric patients with respiratory distress.[30] Abramo et al compared a single EtCO_2 _value to capillary pCO_2 _in pediatrics patients presenting to the emergency department with respiratory emergencies. Their study also found a high correlation between EtCO_2 _and blood gas pCO_2_. (r = 0.87, p= < 0.0001).

Important differences between this prior study and our current study exist. Specifically, there were differences in the severity and underlying causes of respiratory distress in the study populations. 73% of study participants in the prior study were discharged home from the emergency department and the study included patients with signs of upper airway obstruction that may not have had any direct pulmonary involvement. In our study, patients were admitted with moderate to severe respiratory distress secondary to significant lower tract disease. All patients were determined by the admitting physician not to be safe for floor management and therefore admitted to the InCU. Even in this setting, EtCO_2 _was still highly correlated to blood gas pCO_2 _and EtCO_2 _monitoring proved useful in the clinical management of the patients beyond just 'following the trend.'

One of the main objectives of our study was to determine if EtCO_2 _can replace blood gas pCO_2 _in the management of non-intubated pediatric patients with moderate to severe respiratory distress. This decision should be based on the level of agreement between the two methods.[26] Though we found high correlation between EtCO_2 _and venous pCO_2_, correlation is not a measure of agreement but instead is a measure of association. Perfect agreement exists between two methods only when pairs of measurements lie along their line of unity with a slope of 1 and an intercept of 0. Conversely, perfect correlation between two methods exists when pairs of measurements approximate any straight line.[27] For example, if two different scales measuring body weight in kilograms always differed by 20, the two scales would be highly correlated to one another but their level of agreement would be low. High correlation can therefore conceal a significant lack of agreement. Consequently, agreement not correlation determines if one method can replace the other.

The Bland-Altman analysis allows for an overall assessment of agreement between two methods. From the Bland-Altman analysis, the bias ± SD between EtCO_2 _and vpCO_2 _in our study was -3.68 ± 4.70 mmHg. This bias did not vary across the averaged values of EtCO_2 _and vpCO_2_. Abramo et al[30] found a comparable bias between capillary CO_2 _and EtCO_2 _of 3.2 ± 2.4 mmHg. Physiologically, there exists a difference between ideally measured EtCO_2 _concentration and blood gas pCO_2_, whether arterial or venous. Assuming a difference between arterial and mixed-venous pCO_2 _of 2–5 mmHg[31] one would expect at least a 2–5 mmHg difference between EtCO_2 _and venous pCO_2_. Therefore, the observed mean difference of -3.68 closely approximates the true physiologic difference.

The 95% limits of agreement between EtCO_2 _and vpCO_2 _ranged from -12.88 to +5.53 mmHg. This range of EtCO_2 _values is clinically too imprecise for EtCO_2 _to replace vpCO_2 _in the clinical management of patients with respiratory distress. However, knowing the range of EtCO_2 _values can inform clinical decisions in regards to the management of these patients.

In an effort to further define the ability of EtCO_2 _to accurately reflect the true value of vpCO_2_, we found on further analysis that the regression line of EtCO_2 _in Figure 3 was within 2–3 mmHg of the line of unity between EtCO_2 _and vpCO_2 _measurements when vpCO_2 _was below 35 mmHg. At higher vpCO_2 _values, EtCO_2 _was not as accurate. Therefore, for values of vpCO_2 _of 35 mmHg or less, EtCO_2 _may have an acceptable level of accuracy to serve as indirect measurement of vpCO_2 _and thereby be a therapeutic guide.

Our study has several limitations. First, no prior study has used venous pCO_2 _in assessing the accuracy of EtCO_2 _to reflect blood gas pCO_2 _content. Venous pCO_2 _levels reflect many factors beyond just the ventilatory status of the patient including global oxygen consumption, O_2 _delivery, tissue perfusion, level of metabolic acidosis, and hypoxia.[32] Therefore, venous pCO_2 _is not regarded as the gold standard to which EtCO_2 _should be compared.

The use of venous pCO_2 _in this study was determined mainly by our patient population. Arterial blood gas evaluations in non-intubated pediatric patients are only performed when absolutely necessary secondary to their invasive nature, risk of complication, and high level of pain associated with the procedure.[33,34] In the pediatric population, repeat arterial blood gas evaluations tend to be limited to PICU settings in which patients are intubated and sedated. Venous blood gases are therefore more relied upon in assessing the respiratory status of patients in the general pediatric population. Of note, studies comparing venous pCO_2 _and arterial pCO_2 _have found a high level of correlation and agreement between the two measures, especially in patients who have good tissue perfusion. [35-37] The patient population in this study was limited to those patients without signs of vascular compromise or metabolic disease in order to ensure that the venous pCO_2 _values would most accurately reflect the ventilatory status of the patient. Furthermore, by using venous samples for blood gas pCO2 measurement we feel that our study more accurately reflects current practice patterns in the management of pediatrics patients with respiratory distress who are not receiving sedation for invasive ventilatory support

Another limitation of our study was the timing of measurements. Though most evaluations of EtCO_2 _and blood gas pCO_2 _were recorded as simultaneous, a number of paired values were not done instantaneously. We limited the time difference between the two measurements to 10 minutes for inclusion in our study. Under ideal circumstances, blood gas evaluations and EtCO_2 _measurements should be done simultaneously because of the minute to minute fluctuations in CO_2 _levels. A final limitation of the study was its retrospective nature which may have introduced bias.

Despite these several limitations, we still demonstrated a high correlation and moderate amount of agreement between both vpCO_2 _and EtCO_2_. We have also further defined the role of EtCO_2 _in the management of non-intubated pediatric patients with moderate to severe respiratory distress.

## Conclusion

This study demonstrates the utility of EtCO_2 _as a correlate to vpCO_2 _in the clinical assessment of pediatric patients with moderate to severe respiratory distress. Though EtCO_2 _monitoring does not replace blood gas assessment it can serve as an important adjunct in the clinical management of pediatric patients even with significant pulmonary disease. Further study should continue to define the utility of EtCO_2 _as a measure of blood gas pCO_2 _in pediatrics patients with moderate to severe respiratory distress.

## Abbreviations

EtCO_2_: End-tidal Carbon Dioxide; vpCO2: Venous Blood Gas Carbon Dioxide; InCU: Intermediate Care Unit; PICU: Pediatric Intensive Care Unit; ED: Emergency Department; CHB: Children's Hospital Boston; SD: Standard Deviation; SE: Standard Error; OR: odds ratio; CI: confidence interval.

## Competing interests

The authors declare that they have no competing interests.

## Authors' contributions

JM was the principal investigator, largely responsible for data collection, data analysis, manuscript preparation and revision. JA served the role of research assistant, playing a significant role in data analysis and figure preparation. MA was the primary mentor, specifically involved in study design, data analysis, manuscript preparation and revision. All authors read and approved the final manuscript.

## Pre-publication history

The pre-publication history for this paper can be accessed here:


